# ﻿*Formosaniaimmaculata*, a new species of hillstream loach (Teleostei, Cypriniformes, Gastromyzontidae) from the Ou-Jiang River, Southeast China

**DOI:** 10.3897/zookeys.1182.104240

**Published:** 2023-10-16

**Authors:** Wei Sun, Jia-Jun Zhou, Jin-Quan Yang

**Affiliations:** 1 Shanghai Universities Key Laboratory of Marine Animal Taxonomy and Evolution, Shanghai Ocean University, Shanghai 201306, China Shanghai Ocean University Shanghai China; 2 Zhejiang Forest Resource Monitoring Center, Hangzhou 310020, China Zhejiang Forest Resource Monitoring Center Hangzhou China; 3 Zhejiang Forestry Survey Planning and Design Company Limited, Hangzhou 310020, China Zhejiang Forestry Survey Planning and Design Company Limited Hangzhou China

**Keywords:** cytochrome *b*, freshwater fish, key, molecular phylogeny, morphology, taxonomy

## Abstract

*Formosaniaimmaculata*, a new species, is described from the Ou-Jiang basin in Zhejiang Province, Southeast China. It is distinguished from other species of the genus by having a combination of the following characteristics: body without obvious mottling; snout length longer than postorbital length; abdominal scaleless area extending to middle of pectoral-fin base; shorter rostral barbels, the outermost pair length 112.9%–140.0% of eye diameter; and shorter lower lip papillae, length 19.9%–24.4% of eye diameter. Its validity is also affirmed by its distinct *Cytb* gene sequence divergence from all congeners and its monophyly recovered in a *Cytb* gene-based phylogenetic analysis.

## ﻿Introduction

The genus *Formosania* (Cypriniformes: Gastromyzontidae) was formerly known as *Crossostoma* Sauvage, 1878. Because of the junior homonym of *Crossostoma* Morris & Lycett, 1851 (Gastropoda), Novak et al. (2006) suggested the replacement name *Formosania* Oshima, 1919 for the genus, and the type species is *Formosaniadavidi* (Sauvage, 1878). *Formosania* can be distinguished from other genera of the family Gastromyzontidae by having the following characters: snout with a fringe of 13 small barbels; one or two pairs of maxillary barbels; gill opening extending the ventral surface of the head; and dark lateral stripes or blotches on the body (Chen and Tang 2000).

This genus is a group of small loaches endemic to southern China that have adapted to fast-flowing mountain streams and has been considered to be restricted to Fujian and Guangdong Provinces and the west of Taiwan Island (Chen and Tang 2000). However, in the last ten years, two new species have been described from the Ou-Jiang River and the Feiyun-Jiang River in Zhejiang Province, north of Fujian Province (Wang et al. 2006; Zhang and Wang 2011). According to the Catalog of Fishes (https://researcharchive.calacademy.org/research/ichthyology/catalog/fishcatmain.asp), there are nine valid species in the genus, which are *Formosaniadavidi* (Sauvage, 1878), *F.lacustre* (Steindachner, 1908), *F.stigmata* (Nichols, 1926), *F.fascicauda* (Nichols, 1926), *F.tinkhami* (Herre, 1934), *F.paucisquama* (Zheng, 1981), *F.chenyiyui* (Zheng, 1991), *F.fasciolata* (Wang et al., 2006), and *F.galericula* (Zhang, 2011) (Nichols 1926; Chen 1980; Zheng 1981, 1991; Chen and Tang 2000; Wang et al. 2006; Zhang and Wang 2011).

While examining the fish collected from one stream flowing into the Ou-Jiang River in Wuyi County, Zhejiang Province, we found some specimens of *Formosania* that could not be assigned to any described species. Further morphological and molecular analyses of these specimens support them as belonging to a new species described herein.

## ﻿Material and methods

### ﻿Specimen sampling, preservation and morphological analysis

Specimens of the new species were captured in a fish survey conducted in June 2021. Among the 18 collected specimens, five were preserved in 95% ethyl alcohol for DNA extraction, and the remaining 13 specimens were fixed in 10% formalin for two days and then preserved in 70% ethyl alcohol for morphological examination. Two paratype specimens were deposited at the Zhejiang Museum of Natural History, Hangzhou City, Zhejiang Province, and the holotype and the remaining paratypes and alcohol-preserved specimens were deposited at Shanghai Ocean University, Shanghai City, China. Another eight congeneric species of mainland China, which were caught from other fish surveys, were also included for molecular analysis in this study. The three species (*Formosaniadavidi*, *F.galericula* and *F.fasciolata*) were used for morphological comparison with the new species because they are similar in morphology and geographically adjacent. The suffixes -Jiang and -Xi indicate rivers and streams, respectively, in Mandarin Chinese.

All measurements were taken point-to-point with a digital caliper and recorded to the nearest 0.1 mm, following Yi et al. (2014). Measurements and counts were made on the left side of the specimens whenever possible. Morphometric measurements were expressed as percentages of standard length (SL), head length (HL), or eye diameter. The numbers of fin rays and lateral-line scales were counted under a research microscope.

### ﻿DNA extraction, PCR amplification and sequencing

Genomic DNA was extracted from the alcohol-preserved pectoral-fin tip, and the mitochondrial cytochrome *b* (*Cytb*) gene was selected for amplification and sequencing. The *Cytb* gene was amplified by polymerase chain reaction in 25 μL reactions containing 9.5 μL of H_2_O, 1 μL of each primer, 1 μL of template DNA, and 12.5 μL of Taq Master Mix (Sangon Co.,Ltd., Shanghai, China). Polymerase chain reaction (PCR) was performed at 95 °C predenaturing (3 min), then at 94 °C denaturing (30 s), 54 °C annealing (45 s), 72 °C extension (1 min) for 35 cycles, and 72 °C final extension (5 min). The primer pairs used for amplification and sequencing were L14724 (GACTTGAAAAACCACCGTTG) and H15915 (CTCCGATCTCCGGATTACAAGAC) (Xiao et al. 2001). Amplified products were subsequently purified and utilized for sequencing by a commercial sequencing company. The obtained sequences were spliced using Seqman from DNASTAR’s Lasergene (Burland 2000) and then checked by utilizing BLAST analysis in the GenBank database. After confirmation, the targeted sequences were submitted to the GenBank database (Table 1), and provided accession numbers.

**Table 1.** The samples used in this study with their localities, voucher information and GenBank numbers.

Species	River	Sampling localities	Voucher number	GenBank Accession No.	Source
*Formosaniachenyiyui*	Han-jiang	Changting County, Fujian	SHOU20150001	OQ605797	This study
	Han-jiang	Fujian	–	MK135435	Wan et al. 2019
*Formosaniadavidi*	Min-jiang	Qingyuan County, Zhejiang	SHOU202106251	OQ605818	This study
	Min-jiang	Qingyuan County, Zhejiang	SHOU202106252	OQ605819	This study
	Min-jiang	Qingyuan County, Zhejiang	SHOU202106253	OQ605820	This study
	Min-jiang	Qingyuan County, Zhejiang	SHOU202106262	OQ605821	This study
	Min-jiang	Qingyuan County, Zhejiang	SHOU202106263	OQ605822	This study
*Formosaniafascicauda*	Jiulong-jiang	Nanjing County, Fujian	SHOU202201065	OQ605796	This study
	–	–	–	AY392469	Wang 2004
	–	–	–	AY392470	Wang 2004
*Formosaniafasciolata*	Feiyun-jiang	Taishun County, Zhejiang	SHOU202107001	OQ605808	This study
	Feiyun-jiang	Taishun County, Zhejiang	SHOU202107002	OQ605809	This study
	Feiyun-jiang	Taishun County, Zhejiang	SHOU202107003	OQ605810	This study
	Feiyun-jiang	Taishun County, Zhejiang	SHOU202107004	OQ605811	This study
	Feiyun-jiang	Taishun County, Zhejiang	SHOU202107005	OQ605812	This study
*Formosaniagalericula*	Ou-jiang	Qingyuan County, Zhejiang	SHOU202106273	OQ605803	This study
	Ou-jiang	Qingyuan County, Zhejiang	SHOU202106275	OQ605804	This study
	Ou-jiang	Qingyuan County, Zhejiang	SHOU202106276	OQ605805	This study
	Ou-jiang	Qingyuan County, Zhejiang	SHOU202106277	OQ605806	This study
	Ou-jiang	Qingyuan County, Zhejiang	SHOU202106293	OQ605807	This study
New species	Ou-jiang	Wuyi County, Zhejiang	SHOU202106312	OQ605813	This study
	Ou-jiang	Wuyi County, Zhejiang	SHOU202106313	OQ605814	This study
	Ou-jiang	Wuyi County, Zhejiang	SHOU202106314	OQ605815	This study
	Ou-jiang	Wuyi County, Zhejiang	SHOU202106315	OQ605816	This study
	Ou-jiang	Wuyi County, Zhejiang	SHOU202106316	OQ605817	This study
*Formosanialacustre*	–	Taiwan	–	AY392455	Wang 2004
	–	Taiwan	–	AY392456	Wang 2004
	–	Taiwan	–	AY392457	Wang 2004
	–	Taiwan	–	AY392458	Wang 2004
	–	Taiwan	–	AY392459	Wang 2004
*Formosaniapaucisquama*	Lian-jiang	Puning County, Guangdong	SHOU202110011	OQ605798	This study
	Rong-jiang	Jiexi County, Guangdong	SHOU202110028	OQ605799	This study
*Formosaniastigmata*	Min-jiang	Yanping County, Fujian	SHOU202201013	OQ605800	This study
	Min-jiang	Yanping County, Fujian	SHOU202201019	OQ605801	This study
	Min-jiang	Yanping County, Fujian	SHOU202201027	OQ605802	This study
*Formosaniatinkhami*	Zhu-jiang	Longmen County, Guangdong	SHOU202110086	OQ605795	This study
*Vanmaneniastenosoma*	–	–	–	KX056122	GenBank
*Vanmaneniapingchowensis*	–	Wuyuan, Jiangxi	IHCAS0000066	DQ105219	Tang et al. 2006

### ﻿Phylogenetic reconstruction

We sequenced 28 *Cytb* gene sequences of *Formosania* and retrieved 8 *Cytb* gene sequences of *Formosania* from GenBank. *Vanmaneniastenosoma* and *V.pingchowensis* were selected as outgroups for molecular phylogeny analysis (Table 1). A multiple sequence alignment was prepared for all sequences using MEGA v.11.0 (Tamura et al. 2021). The genetic distances (p-distance with 1000 bootstraps) of the sequences among taxa were also calculated by using MEGA v.11.0. The best substitution models (TIM2+R3) for maximum likelihood (ML) and the best substitution model (GTR+G+I) for Bayesian inference (BI) were selected in ModelFinder (Kalyaanamoorthy et al. 2017) by Akaike’s information criterion (AIC). The phylogenetic trees were inferred using Bayesian inference (BI) and maximum likelihood (ML) approaches. Bayesian analyses were conducted using MrBayes (Ronquist et al. 2012). Four simultaneous Monte Carlo Markov chains were run for 2 million generations, with sampling one tree per 100 replicates for each run, and the first quarter of the trees were discarded as burn-in; the remaining trees from two independent runs were used to construct a consensus tree. The ML analyses were conducted using IQ-TREE (Nguyen et al. 2015) with a total of 20 000 bootstrap replications performed.

## ﻿Results

### ﻿Taxonomic account

**Family Gastromyzontidae Hora,1950**

#### ﻿Genus *Formosania* Oshima,1919

##### ﻿ Formosania immaculata

Taxon classificationAnimaliaCypriniformesGastromyzontidae

Sun, Zhou & Yang sp. nov.

3B11900F-CA04-5EF8-8D7B-C3278F4771FB

https://zoobank.org/E62FDC2E-148C-45B3-8BC1-2AD18C2B486A

Figs 1 , 2A , 5

###### Type material.

***Holotype*.** SHOU2021060325, 87.9 mm total length (TL), 77.4 mm standard length (SL), adult collected by Jia-Jun Zhou and Wei Sun on June 28, 2021, in Wuyi County, Jinhua City, Zhejiang Province, China (28.7179°N, 119.4939°E; c. 882 m a.s.l.).

***Paratypes*.** Twelve specimens from the same locality as the holotype, SHOU2021060326-060337, 43.2–68.7 mm SL, were collected by Wei Sun and Jia-Jun Zhou on June 28, 2021.

###### Description.

**Figure 1.**
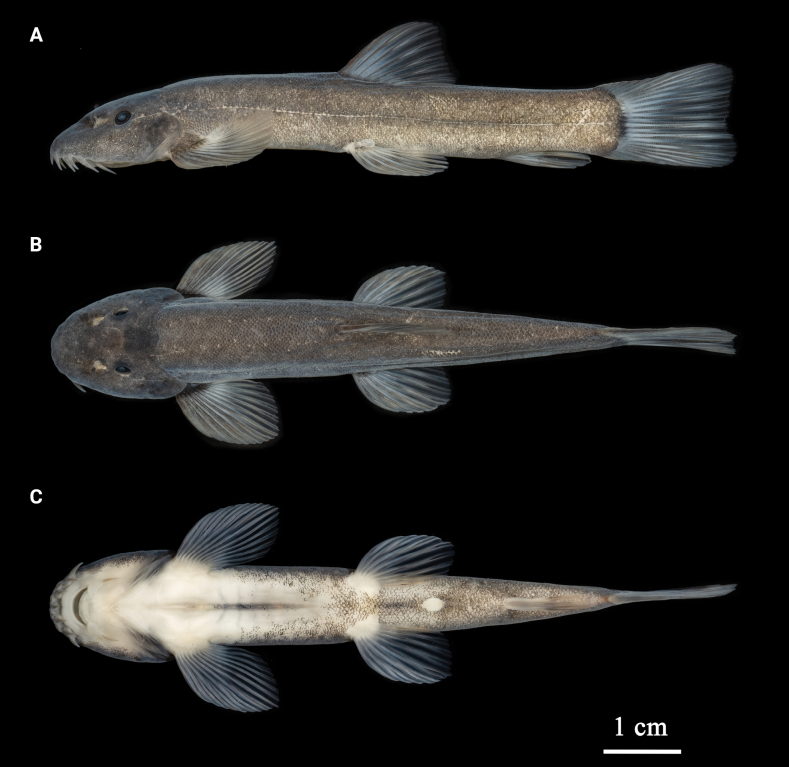
Lateral (**A)** dorsal (**B)** and ventral (**C)** views of *Formosaniaimmaculata* sp. nov., holotype, adult, SHOU2021060325.

**Figure 2.**
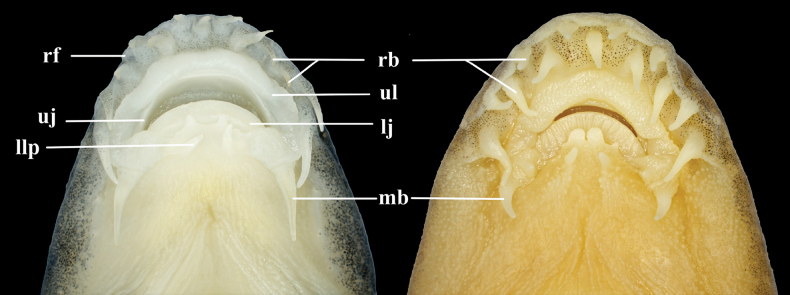
Ventral view of mouth of **A***Formosaniaimmaculata* sp. nov., SHOU2021060325, holotype **B***Formosaniastigmata*, SHOU2021060180. lj: lower jaw; llp: lower lip papilla; mb: maxillary barbel; rb: rostral barbel; rf: rostral fold; uj: upper jaw; ul: upper lip.

Morphometric measurements for the specimens examined are given in Table 2. See Fig. 1A–C for lateral, dorsal, and ventral views of the body and Fig. 2A for its mouthpart structures.

**Table 2.** Morphometric measurements and meristic counts for *Formosaniaimmaculata* sp. nov., *F.davidi*, *F.fasciolata* and *F.galericula*.

Characters	*F.immaculata* sp.nov. (*N*=13)			*F.davidi* (*N*=15)		*F.fasciolata* (*N*=8)		*F.galericula* (*N*=11)	
	Holotype	Holotype+paratypes							
		Range	Mean+SD	Range	Mean+SD	Range	Mean+SD	Range	Mean+SD
Standard length (mm)	77.4	43.2–77.4	61.7±7.80	58.5–75.4	67.8±6.08	50.4–73.4	58.9±7.85	50.5–67.3	59.0±4.63
% **of standard length (SL)**									
Body depth	17.9	16.1–21.1	17.6±1.29	17.3–19.2	18.4±0.59	16.2–17.4	16.8±0.41	13.5–18.4	15.5±1.37
Head length	22.9	22.5–25.9	23.6±1.05	21.1–24.5	23.1±0.99	22.7–27.0	24.2±1.48	22.7–26.3	24.5±1.00
Head depth	12.6	11.5–13.9	12.7±0.72	11.5–13.4	12.5±0.55	12.5–13.4	13.0±0.29	11.8–13.3	12.5±0.48
Head width	18.7	17.4–19.7	18.3±0.61	16.0–19.5	17.8±1.07	18.1–19.8	19.0±0.61	13.5–20.0	18.0±1.99
Length of caudal peduncle	12.2	12.2–14.3	13.2±0.65	10.5–13.9	11.8±1.01	10.2–12.8	11.1±0.82	10.4–13.0	11.8±0.83
Depth of caudal peduncle	12.4	12.4–14.3	12.9±0.59	11.3–13.2	12.2±0.58	12.2–13.8	13.1±0.56	10.2–12.7	11.3±0.65
Dorsal-fin length	20.7	19.6–22.4	20.9±0.90	20.2–23.1	21.4±0.96	20.2–23.2	21.8±1.03	19.6–22.4	21.2±0.84
Pectoral-fin length	23.5	22.0–25.0	23.7±0.92	22.3–24.2	23.3±0.53	22.8–27.4	24.5±1.47	22.4–26.7	25.0±1.31
Pelvic-fin length	20.3	19.0–21.2	19.9±0.65	19.2–21.6	20.2±0.80	19.8–22.2	21.0±0.90	19.1–21.8	20.8±0.87
Anal-fin length	19.1	16.8–19.1	18.1±0.74	17.7–20.5	19.3±1.04	18.1–22.1	19.6±1.18	17.1–19.8	18.4±0.99
Dorsal-fin base length	12.6	12.3–13.8	12.8±0.46	11.3–14.1	12.8±0.85	12.6–15.3	13.7±0.91	10.5–13.6	12.4±0.83
Pectoral-fin base length	7.5	6.6–8.4	7.3±0.58	6.2–8.0	6.8±0.57	6.3–8.1	7.4±0.61	6.4–8.5	7.3±0.58
Pelvic-fin base length	5.1	4.6–5.4	5.1±0.26	4.3–5.3	4.7±0.32	4.4–5.7	5.2±0.44	4.6–5.5	5.2±0.29
Anal-fin base length	7.4	6.4–8.1	7.1±0.51	6.3–8.0	7.3±0.59	5.9–8.6	7.2±1.03	6.0–7.7	6.9±0.49
Predorsal length	47.5	45.4–50.5	48.2±1.45	47.3–49.9	49.0±0.96	46.7–51.2	48.8±1.37	47.6–49.8	49.1±0.72
Prepectoral length	18.2	18.1–20.9	19.5±0.96	17.2–20.8	19.3±1.00	20.2–22.2	21.0±0.69	17.8–23.1	20.7±1.36
Prepelvic length	52.1	50.8–54.5	52.8±1.07	51.7–55.0	53.4±0.94	52.2–54.9	53.8±0.95	52.0–55.8	54.2±1.42
Preanal length	79.3	78.1–80.4	79.4±0.74	77.2–82.8	79.9±1.91	78.4–83.3	80.6±1.71	75.9–82.2	79.8±1.94
% **of head length (HL)**									
Snout length	47.8	44.6–48.2	46.0±0.93	40.7–44.8	43.2±1.35	42.2–43.8	43.2±0.49	39.9–45.9	43.1±1.96
Head depth	55.0	48.7–60.6	53.9±3.44	50.1–56.9	53.8±2.17	49.5–58.8	53.8±3.08	47.6–53.9	51.0±1.99
Eye diameter	13.9	13.7–16.8	15.1±1.07	13.1–15.8	14.3±0.90	14.8–17.7	16.3±0.92	15.1–17.3	15.9±0.81
Interorbital width	43.4	35.1–43.4	39.2±2.53	32.1–42.5	38.8±2.73	32.6–41.9	37.9±2.90	33.3–39.1	36.4±1.88
% **of caudal peduncle length**									
Depth of caudal peduncle	101.7	91.0–101.9	97.8±3.74	86.0–109.3	103.4±7.34	107.9–130.6	118.8±7.85	86.2–106.2	96.5±6.01
% **of eye diameter**									
the outermost pair of rostral barbels length	124.2	112.9–140.0	122.2±8.61	128.3–175.4	159.0±15.63	119.8–167.5	145.6±14.01	105.6–138.8	113.5±9.09
Maxillary barbels length	113.7	94.2–123.9	105.1±9.00	94.3–150.4	126.9±17.61	94.0–142.0	120.1±14.25	83.5–107.0	95.0±7.01
Lower lip papillae length	23.2	19.9–24.4	22.8±1.59	47.0–61.9	53.0±5.36	40.3–51.0	47.0±3.25	16.3–25.4	21.0±3.02
% **of the postorbital length**									
Snout length	124.3	114.5–125.0	119.6±3.62	97.7–102.7	100.6±1.46	99.6–103.5	101.6±1.43	96.4–102.4	99.3±1.78
**Meristic counts**									
Dorsal-fin rays	iii,8	iii,8		iii,8		iii,8		iii,8	
Pectoral-fin rays	i,14	i,13–14		i,14–15		i,14		i,13–14	
Pelvic-fin rays	i,8	i,8		i,8		i,8		i,8	
Anal-fin rays	ii,5	ii,5		ii,5		ii,5		ii,5	
Lateral-line scales	92	92–98		89–100		90–96		89–95	

Head depressed in lateral view; head width always greater than depth; head width 17.4–19.7% of SL. Snout obtuse in dorsal view and longer than postorbital length; snout length 114.5%–125.0% of postorbital length. Mouth inferior and arched. Lips fleshy, with upper lip wide, without obvious convex particles; lower lip with a pair of papillae and a pair of lobulated papillae (Fig. 2A). Upper lip connected to lower lip around the corners of mouth by a papillated flap and one pair of maxillary barbels at the corners of mouth. Upper jaw covered by upper lip. Rostral fold appeared at end of snout, with 13 well-developed rostral barbels in an irregular row, covered with small spots. All rostral barbels connected with rostral fold. Outermost pair of rostral barbels longest and slightly longer than eye diameter, with a length of 112.9%–140.0% of eye diameter. Anterior and posterior nostrils adjacent with a well-developed flap on anterior ones. Eyes normal; diameter 13.7%–16.8% of HL. Gill openings reached ventral surface of head, with its upper extremity reaching the level of upper margin of orbit. Body elongated, the anterior part of body cylindrical and laterally compressed behind dorsal-fin base. The greatest depth of body at dorsal-fin origin and the least depth at caudal-fin base; body depth at dorsal-fin origin 16.1%–21.1% of SL. Body scaled but scales absent on head and before the middle of pectoral-fin base of abdomen. Scales minute, lateral line complete with 92–98 perforated scales. Caudal peduncle compressed laterally; length equal to peduncle depth.

Dorsal fin had three unbranched and eight branched rays; origin slightly in front of pelvic-fin insertion, situated slightly ahead to the midpoint between snout tip and caudal-fin base. Pectoral fin developed, with one unbranched and 13–14 branched rays. Pelvic fins long with one unbranched and eight branched rays, tips of depressed pelvic fins reaching anus when pelvic-fin rays extended backward. Anus in middle of pelvic-fin insertion and anal-fin insertion; anal fin with two unbranched and five branched rays, with the tip of anal fin closing or reaching to caudal-fin base. Caudal fin slightly forked; lower lobe slightly longer than upper lobe.

***Coloration*.** In life, body slightly brown; fins and rostral barbels slightly red; with inconspicuous black blotches on back of head (Fig. 5). In 10% formalin-fixed specimens, dorsal and flank of head and body grayish-brown; ventral surface of head and abdomen white to yellowish with many black spots after pectoral fins; all fins hyaline and light gray, without obvious blotches (Fig. 1).

###### Diagnosis.

**Figure 3.**
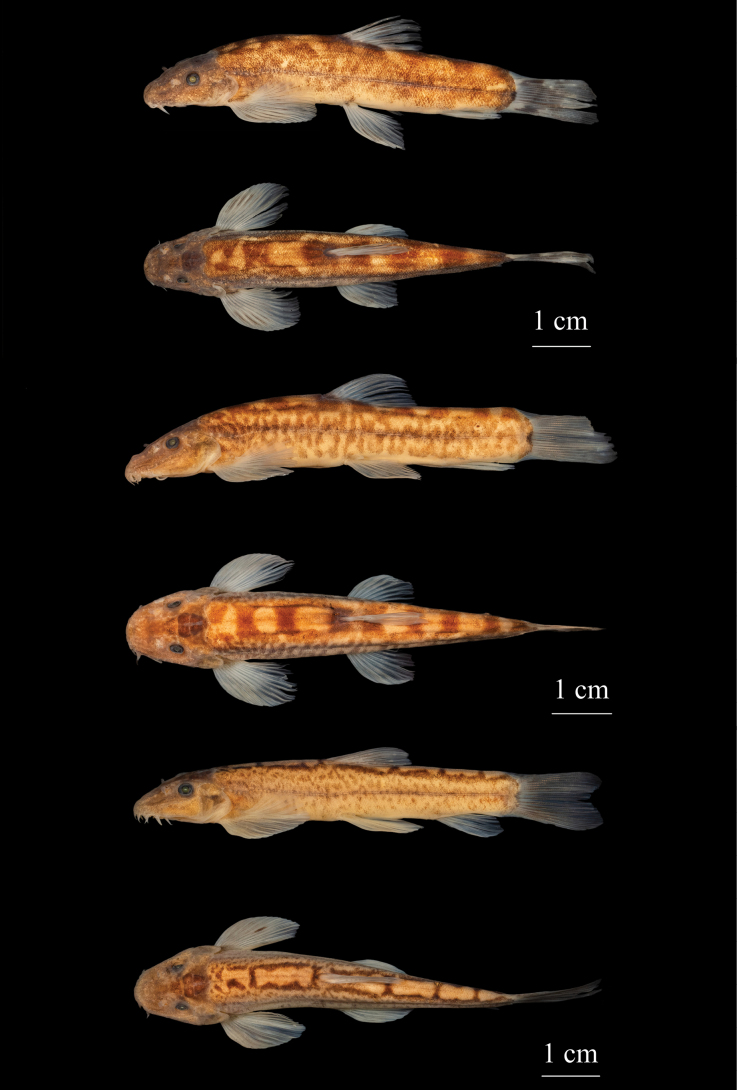
Lateral and dorsal views of **A***Formosaniadavidi*, SHOU2021060176 **B***Formosaniafasciolata*, SHOU2021060200 **C***Formosaniagalericula*, SHOU2021060169.

*Formosaniaimmaculata* sp. nov. resembles the *Formosaniadavidi* species group (*F.davidi*, *F.galericula* and *F.fasciolata*) in having 13 well-developed rostral barbels arranged in one irregular row (Fig. 2A), while other congeners arranged in 2 rows (Fig. 2B). It is distinguished from the three species in the *Formosaniadavidi* species group by having no obvious blotches or stripes (vs. having blotches or stripes) on the body and snout length longer than (vs. equal to) postorbital length (see Fig. 3 and Table 3). The new species differs from *F.davidi* in having shorter rostral barbels [outermost pair 112.9%–140.0% (average 122.2) vs. 128.3%–175.4% (average 159.0%) of eye diameter], shorter lower lip papillae [19.9%–24.4% (average 22.8) vs. 47.0%–61.9% (average 53.0%) of eye diameter] and narrower abdominal scaleless area (extending to middle of pectoral-fin base vs. extending slightly behind pectoral-fin axil) (see Table 3); from *F.fasciolata* in having shorter rostral barbels [outermost pair 112.9%–140.0% (average 122.2) vs. 119.8%–167.5% (average 145.6%)] of eye diameter, shorter lower lip papillae [19.9%–24.4% (average 22.8) vs. 40.3%–51.0% (average 47.0%) of eye diameter] and shorter depth of caudal peduncle [91.0%–101.9% (average 97.8%) vs. 107.9%–130.6% (average 118.8%) of caudal peduncle length]; and from *F.galericula* in having narrower abdominal scaleless area (extending to middle of pectoral-fin base vs. extending slightly behind pectoral-fin axil).

**Table 3.** Comparison of characters among *Formosaniaimmaculata* sp. nov., *F.davidi*, *F.fasciolata* and *F.galericula*.

	*F.immaculata* sp. nov.	*F.davidi*	*F.fasciolata*	*F.galericula*
Distribution	Ou-jiang	Min-jiang	Feiyun-jiang	Ou-jiang
Blotches on the back	Absent	7–9 black blotches	7–9 light-colored blotches	7–13 black blotches
Blotches on the flank	Absent	Irregular blotches	18–22 anomalistic stripes	Filled with irregular blotches
Pectoral-fin rays	i,13–14	i,14–15	i,14	i,13–14
Outermost pair of rostral barbels length (% of eye diameter)	112.9–140.0 (122.2±8.61)	128.3–175.4 (159.0±15.63)	119.8–167.5 (145.6±14.01)	105.6–138.8 (113.5±9.09)
Lower lip papillae length (% of eye diameter)	19.9–24.4 (22.8±1.59)	47.0–61.9 (53.0±5.36)	40.3–51.0 (47.0±3.25)	16.3–25.4 (21.0±3.02)
Depth of caudal peduncle (% of caudal peduncle length)	91.0–101.9 (97.8±3.74)	86.0–109.3 (103.4±7.34)	107.9–130.6 (118.8±7.85)	86.2–106.2 (96.5±6.01)
Abdominal scaleless area	Extending to the middle of the pectoral-fin base	Extending slightly behind the pectoral-fin axil	Extending to the middle of the pectoral-fin base	Extending slightly behind the pectoral-fin axil
Snout length (% of the postorbital length)	114.5–125.0 (119.6±3.62)	97.7–102.7 (100.6±1.46)	99.6–103.5 (101.6±1.43)	96.4–102.4 (99.3±1.78)

###### Etymology.

The specific epithet is the Latin form of the word *immaculate* here referring to the unique body of no blotches or stripes. We propose the Chinese common name Wú Bān Yīng Kǒu Qiū (无斑缨口鳅).

###### Distribution and habitat.

**Figure 4.**
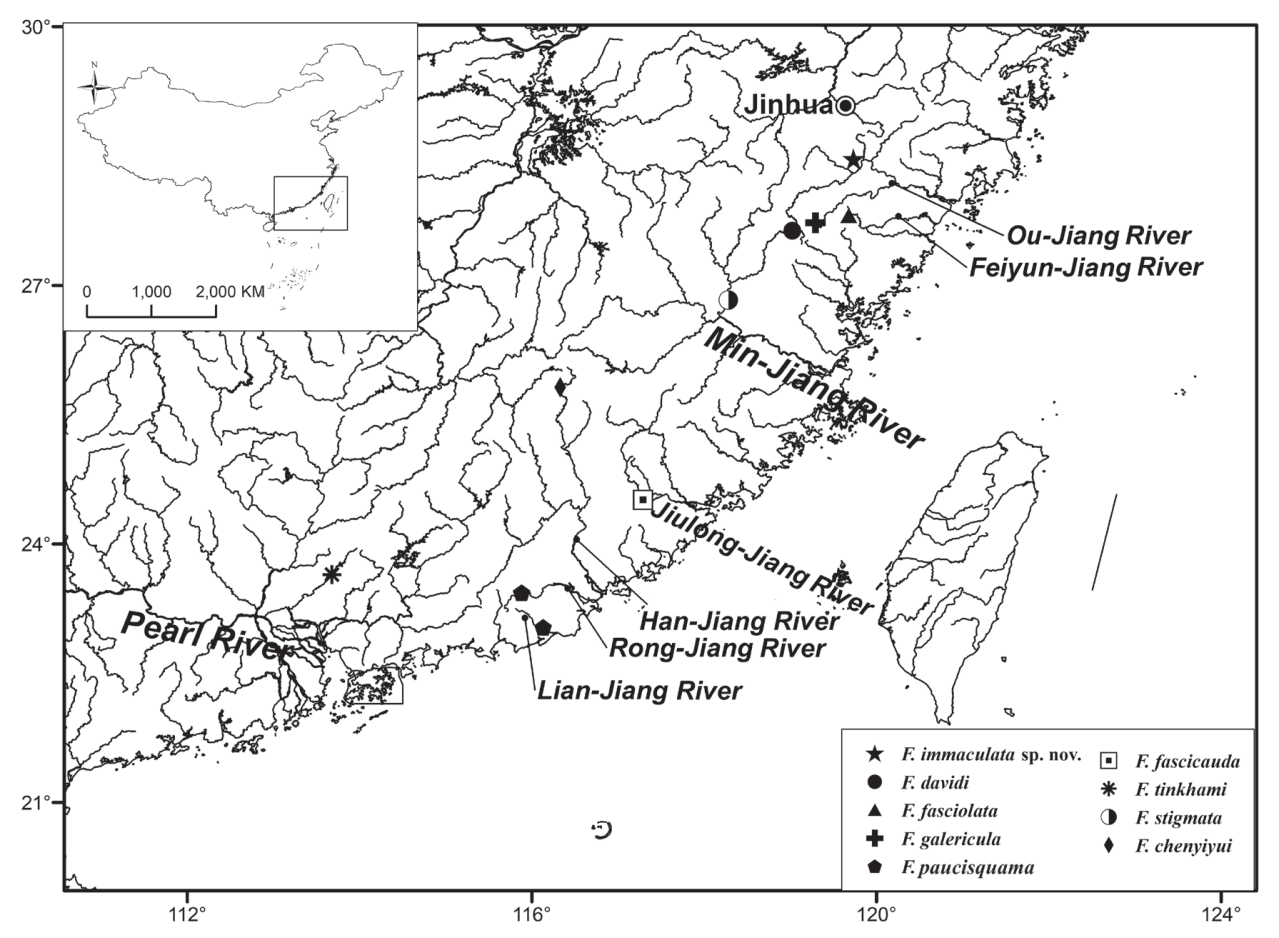
Map showing collection localities of nine species of *Formosania* involved in the present study. The names of rivers are italicized, and the city of Jinhua is highlighted.

**Figure 5.**
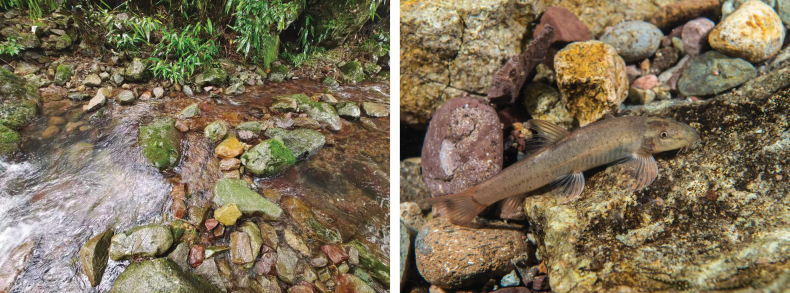
Habitat and live specimen of *Formosaniaimmaculata* sp. nov.

The new species is known only from the upper reaches of the Xuanping-Xi, a stream tributary to the Ou-Jiang River, in Wuyi County, Zhejiang Province, China (Fig. 4). It inhabits fast-flowing streams with gravelly and pebbly substrates (Fig. 5).

### ﻿Molecular analysis

Thirty-six *Cytb* gene sequences of *Formosania* were used for phylogenetic analysis. After alignment and trimming, 1141 bp (base pairs) of the *Cytb* gene was obtained. There were 854 conserved sites, 287 variable sites, 15 singleton sites, and 272 parsimony-informative sites. The mean frequency of four nucleotides was A=25.8%, T=28.2%, C= 30.0%, and G=16.0%; the base composition was A-T rich (54.0%).

**Figure 6.**
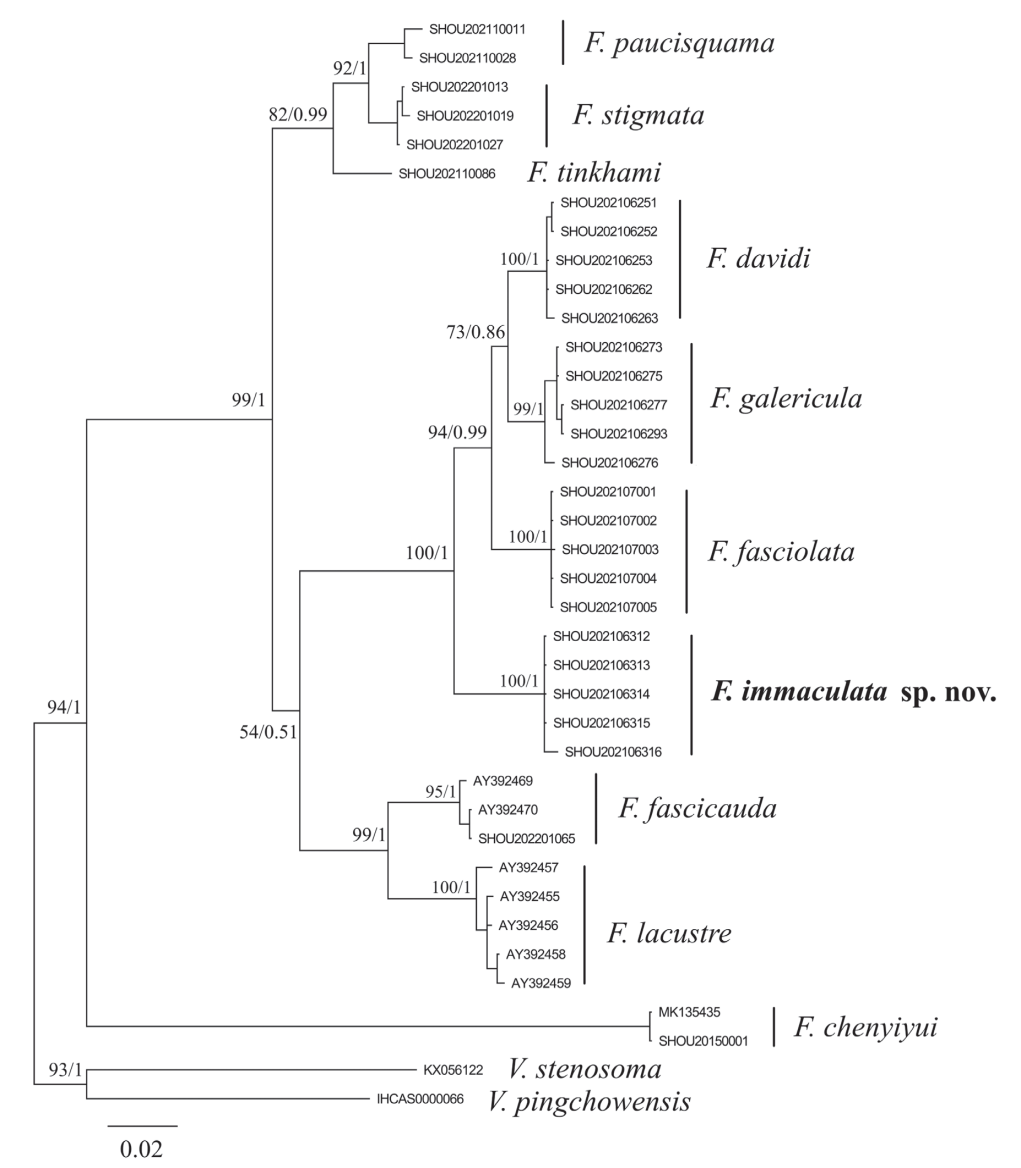
Bayesian inference tree based on mitochondrial *Cytb* gene sequences of 10 *Formosania* species. Maximum likelihood and Bayesian inference analyses resulted in congruent trees. Bootstrap and posterior probability values are shown beside nodes on the tree if 50% or higher.

The two phylogenetic analysis methods (BI and ML) showed an identical topology (Fig. 6). The monophyly of the genus was well supported (94% bootstrap value and 100% posterior probability). Both phylogenetic trees revealed that *Formosaniachenyiyui* is sister to the remaining species of *Formosania*. The remaining nine species formed a monophyletic clade with strong support and then separated into three groups. Samples of *F.immaculata* sp. nov. were monophyletic and belonged to a group of species with robust support (100% bootstrap value and 100% posterior probability). This group is defined here as the *F.davidi* species group, and our molecular data suggest that it includes *F.davidi*, *F.galericula* and *F.fasciolata*.

*Formosaniaimmaculata* sp. nov. had minimal genetic distance with the three similar species, 4.5% with *F.davidi*, 4.5% with *F.galericula*, and 4.7% with *F.fasciolata*, which was greater than the genetic distance among the three similar species (2.5%–3.0%) (Table 4). The mean genetic distance of the new species from all sampled species was 8.6%, far greater than the minimum distance (2.3%), detected here between *F.stigmata* and *F.paucisquama*. *Formosaniachenyiyui* has the greatest genetic divergence from all other species (16.2%–19.1%).

**Table 4.** Genetic distances of *Cytb* computed by MEGA among 10 species of *Formosania*.

	1	2	3	4	5	6	7	8	9
*F.immaculata* sp. nov.									
*F.davidi*	0.045								
*F.galericula*	0.045	0.025							
*F.fasciolata*	0.047	0.030	0.029						
*F.stigmata*	0.091	0.089	0.090	0.091					
*F.paucisquama*	0.089	0.095	0.092	0.093	0.023				
*F.fascicauda*	0.093	0.091	0.091	0.094	0.070	0.071			
*F.lacustre*	0.094	0.092	0.090	0.095	0.074	0.077	0.045		
*F.tinkhami*	0.085	0.088	0.091	0.092	0.031	0.036	0.068	0.074	
*F.chenyiyui*	0.189	0.191	0.184	0.186	0.165	0.172	0.184	0.173	0.162

## ﻿Discussion

*Formosania* species usually inhabit hill streams with relatively fast-flowing currents. Except for *F.fascicauda* and *F.stigmata* and even *F.lacustre*, the rest of the species are limited in distribution, only being found in a single river or a few adjacent rivers (Chen and Tang 2000; Tang and Chen 2000; Wang et al. 2006; Teng 2010; Zhang and Wang 2011). *Formosaniaimmaculata* sp. nov. is known only from the upper reaches of the Xuanping-Xi, a northern stream tributary of the Ou-Jiang River, in Wuyi County, Zhejiang Province, Southeast China, which is currently the northernmost species of *Formosania*. Another species, *F.galericula*, also occurs in the same river system, but it is only found in some southern tributaries of the Ou-Jiang River. *Formosaniafasciolata* is known from the Feiyun-Jiang basin, adjacent to southern the Ou-Jiang River. The last similar species, *F.davidi*, is only distributed in the Min-Jiang River system, a close neighbor of the Feiyun-Jiang River. The new species can be assigned to the *F.davidi* group by sharing 13 rostral barbels in one irregular row, in addition to its distribution in the same or adjacent water systems.

In addition, the new species can be easily distinguished from the *Formosaniadavidi* species group and the rest of the congeneric species by lacking blotches or stripes on the back or flank. In terms of morphometric characteristics, *F.galericula* is most similar to the new species. However, *F.immaculata* sp. nov. can be distinguished from *F.galericula* in possessing a longer snout (114.5–125.0 vs. 96.4%–102.4% of postorbital length) and narrower abdominal scaleless area (extending to middle of pectoral-fin base vs. extending slightly behind pectoral-fin axil) (Table 3).

The validity of *Formosaniaimmaculata* sp. nov. is confirmed by its significant genetic divergence from congeners (Table 4). It has significant genetic distance from other congeners (4.5%–18.9%), far greater than the minimum calculated here between *F.stigmata* and *F.paucisquama* (2.3%), and greater than the genetic distance among the three species in the same group (2.5%–3.0%).

The validity of *Formosaniaimmaculata* sp. nov. is also confirmed by its monophyly in the phylogenetic analysis based on the *Cytb* gene (Fig. 6). There are no reports on the complete phylogenetic and phylogeographic studies of this genus at present. Only Wang et al. (2007) and Teng (2010) have conducted a phylogeographic study on three or four species, which did not include any species in *F.davidi* group. Our results suggest that it should be reasonable for the new species to be designated to the *F.davidi* group for the aforementioned morphological and geographical reasons, which indicates that they originated from a recent common ancestor.

### ﻿Diagnostic key to species of *Formosania*

**Table:** 

1	Rostral barbels number unstable, ranging from 12 to 15, their length less than half of the eye diameter; two lengthwise-ribbon-like stripes on back, one in front of the dorsal-fin axil and one behind	***F.chenyiyui* (Ting-Jiang River)**
–	Rostral barbels number stable, always 13, their length great than half of the eye diameter; saddle-like or irregular stripes on back	**2**
2	Rostral barbels arranged in two rows, the front row on top of the rostral fold, the back row in the center of the rostral groove; two pairs of maxillary barbels, the inside pair are papillae	**3**
–	Rostral barbels arranged in an irregular row, the base of which connected with the rostral fold; one pair of maxillary barbels	**7**
3	Cloud-like stripes on side	**4**
–	Wavy-longitudinal-like stripes or blotches on side	**6**
4	Caudal peduncle stout, its depth greater than its length	***F.lacustre* (Mulan-Xi and Jin-Jiang Rivers, Taiwan Island)**
–	Caudal peduncle slender, its depth less than or equal to its length	**5**
5	86–105 perforated scales; narrower abdominal scaleless area extending slightly behind the pectoral-fin axil	***F.stigmata* (From Min-jiang to Han-jiang Rivers)**
–	76–83 perforated scales; narrower abdominal scaleless area extending to the middle of pectoral-fin base	***F.paucisquama* (Lian-Jiang, Rong-Jiang and Han-Jiang Rivers)**
6	Several black-and-white and wavy-longitudinal-like stripes on side; 6–8 saddle-like stripes on back	***F.fascicauda* (Jiulong-Jiang River)**
–	Two or three rows of blotches on side; 5–7 irregular stripes on back	***F.tinkhami* (Pearl River)**
7	No obvious blotches or stripes on body; snout length 1.2 times greater than the postorbital length	***F.immaculata* sp. nov. (Ou-Jiang River)**
–	With obvious blotches or stripes on body; snout length equal to the postorbital length	**8**
8	Caudal peduncle stout, its depth greater than its length; 18–22 anomalistic stripes on side; narrower abdominal scaleless area extending to the middle of pectoral-fin base	***F.fasciolata* (Feiyun-Jiang and Ou-Jiang Rivers)**
–	Caudal peduncle slender, its depth less than or equal to its length; with irregular blotches on side; narrower abdominal scaleless area extending slightly behind the pectoral-fin axil	**9**
9	Length of longest rostral barbel about 1.5 times greater than the eye diameter; 7–9 saddle-like stripes on back	***F.davidi* (Min-Jiang River)**
–	Length of longest rostral barbel equal to the eye diameter; 7–13 irregular stripes on back	***F.galericula* (Ou-Jiang River)**

### ﻿Comparative materials

*Formosaniadavidi*: SHOU2021060096-106, SHOU2021060176-179, 15, 58.5–75.4 mm SL; Qingyuan County, Min-Jiang River System, Zhejiang Province, China.

*Formosaniafascicauda*: SHOU202201083-091, 9, 53.4–69.1 mm SL; Nanjing County, Jiulong-Jiang River System, Fujian Province, China.

*Formosaniafasciolata*: SHOU2021060193-200, 8, 50.8–73.4 mm SL; Liguang stream in Wuyanling National Nature Reserve (type locality), Taishun County, Feiyun-Jiang River System, Zhejiang Province, China.

*Formosaniagalericula*: SHOU2021060165-175, 11, 50.5–67.4 mm SL; unnamed stream in Hehu village (type locality), Qingyuan County, Ou-Jiang River System, Zhejiang Province, China.

*Formosaniapaucisquama*: SHOU202110011-013, 4, 50.3–64.4 mm SL; unnamed stream in Da’nan Mountain (type locality), Puning County, Lian-Jiang River System, Guangdong Province, China.

*Formosaniastigmata*: SHOU2021060180-183, 4, 56.0–84.8 mm SL; Qingyuan County, Min-Jiang River System, Zhejiang Province, China.

*Formosaniatinkhami*: SHOU202110086-090, 5, 44.6–58.1 mm SL; unnamed stream in Nankun Mountain (type locality), Longmen County, Pearl River System, Guangdong Province, China.

## Supplementary Material

XML Treatment for Formosania immaculata

## ﻿Additional information

### Author ORCIDs

Jia-Jun Zhou https://orcid.org/0000-0003-1038-1540

Jin-Quan Yang https://orcid.org/0000-0003-0387-1824

### Data availability

All of the data that support the findings of this study are available in the main text.
